# Thyroid glands from pigs with cystic fibrosis, old issues new ways

**DOI:** 10.1113/expphysiol.2010.055079

**Published:** 2010-12

**Authors:** Bob J Scholte

Cystic fibrosis (CF) is a prominent inherited disease in the clinic and in biological science. A frequency near one in three-thousand newborn in the Caucasoid population and a severely reduced lifespan with high morbidity stimulates a continuing effort to study and cure the disease. Cystic fibrosis is caused by mutations in the *CFTR* gene, which encodes a chloride ion channel active primarily in the apical membrane of secretory epithelia. In CF patients, deficient CFTR channel activity results in a lack of chloride secretion and coupled osmotic water transport in secretory epithelia. This causes a complex multi-organ disease involving mainly the airways, pancreas and intestine. Despite an intensive research campaign in the past two decades, no ‘magic bullet’ type of cure for this devastating disease has been found. One of the problems facing the CF researcher has been to generate models that emulate the complexities of the different tissues affected by CF pathology. Cell culture and mouse models have been very instructive but do have serious limitations. Recently, the development of CFTR-deficient pigs (Rogers *et al.* 2008; Stoltz *et al.* 2010) and ferrets (Sun *et al.* 2010) by the teams of Michael Welsh and John Engelhardt of Iowa university have provided new and exciting options. In this issue of *Experimental Physiology*, Hui Li *et al*. (2010) seize this opportunity to study the thyroid gland in the CF pig model, a somewhat neglected but potentially important tissue in CF pathology.

The authors show that cultured thyroid epithelium from *CFTR*-knockout pigs lacks the signature short-circuit current response to the cAMP agonist isoprenaline. This is evidence that CFTR is active as a regulated apical chloride conductance in this tissue. Furthermore, they show that the amiloride-sensitive sodium current, most probably carried by apical *ENaC* type sodium ion channels, is enhanced in mutant epithelium compared with control epithelium, as in CF airways. In addition, they present a model in which the secretion of iodide ions through the Cl^−^–I^−^ exchange carrier SLC26A4 depends on CFTR activity. This would explain the subclinically reduced thyroid function reported in CF patients, which in part relies of the availability of iodide in the thyroid follicles for the production of thyroid hormone. This study confirms the power of the CF pig model and opens new ways to study thyroid function.

Like all seminal papers, it raises questions as well. The hypothetical CFTR-dependent iodide fluxes have not been measured directly in this system yet. This will further substantiate the claim. Furthermore, reduced production of thyroid hormone, subclinically or otherwise, is not yet established in the pig model. Previously, in a paper duly cited by the authors, Devuyst *et al.* (1997) reported expression of CFTR mRNA and antigen in normal human thyroid epithelial cells, and also observed a correlation between the number of epithelial cells positive for CFTR antigen and follicle size. However, the study by Hui Li *et al*. (2010) does not indicate a difference in follicle size between normal and CFTR-deficient pigs. Furthermore, CFTR immunohistochemistry showing the cellular and subcellular localization of CFTR in pig thyroid gland is not presented yet. The CFTR-deficient pig model presents an ideal opportunity to do such studies and settle longstanding issues about the quality and specificity of CFTR antibody staining in tissues expressing low levels of CFTR. The vast majority of CF patients produce a mutant form of CFTR with more or less residual activity, making a true negative control impossible. Another issue is the analysis of thyroid function in the different mutant CFTR mouse models and in the recently developed ferret model. This would establish whether the observed CFTR-related thyroid epithelial transport deficiency is a cross-species phenomenon in mutant animals. The physiological consequences of this deficiency also need to be further examined in these models, and most importantly re-examined in patients.

The near doubling of life expectancy for CF patients since the eighties has been a result of gradually improving the intensive clinical surveillance and treatment of the patients, rather than spectacular breakthroughs in basic science. However, studies in relevant cellular and animal model systems did contribute significantly by generating new insights, and provided testing grounds for novel concepts and therapeutics. In fact, the current pipeline is filled with promising candidates, ranging from anti-inflammatory agents to compounds correcting mutant CFTR dysfunction. The focus on progressive CF airway disease is certainly justified in view of its relative contribution to the burden of CF pathology. However, recent studies, including the one discussed here, have shown that other tissues and cell types also deserve attention. Bile duct, gall bladder, kidney and salivary glands show CFTR-dependent function as well. Non-epithelial cell types, including smooth muscle cells, bone- and cartilage-forming cells and cells of the immune system, express CFTR, and are likely to contribute to CF pathology in a way that has not been clearly defined. The large animal models for CF significantly add to our ability to study known and novel aspects of the disease.
